# Ocrelizumab associates with reduced cerebrospinal fluid B and CD20^dim^ CD4^+^ T cells in primary progressive multiple sclerosis

**DOI:** 10.1093/braincomms/fcae021

**Published:** 2024-01-29

**Authors:** Fabiënne van Puijfelik, Katelijn M Blok, Romy A M Klein Kranenbarg, Jasper Rip, Janet de Beukelaar, Annet F Wierenga-Wolf, Beatrijs Wokke, Marvin M van Luijn, Joost Smolders

**Affiliations:** 1 Department of Immunology, MS Center ErasMS, Erasmus MC, University Medical Center Rotterdam, 3015 CN, Rotterdam, The Netherlands; Department of Neurology, MS Center ErasMS, Erasmus MC, University Medical Center Rotterdam, 3015 GD, Rotterdam, The Netherlands; Department of Neurology, Albert Schweitzer Hospital, 3318 AT, Dordrecht, The Netherlands; Department of Neurology, MS Center ErasMS, Erasmus MC, University Medical Center Rotterdam, 3015 GD, Rotterdam, The Netherlands; Department of Neurology, Albert Schweitzer Hospital, 3318 AT, Dordrecht, The Netherlands; 1 Department of Immunology, MS Center ErasMS, Erasmus MC, University Medical Center Rotterdam, 3015 CN, Rotterdam, The Netherlands; Department of Neurology, Albert Schweitzer Hospital, 3318 AT, Dordrecht, The Netherlands; 1 Department of Immunology, MS Center ErasMS, Erasmus MC, University Medical Center Rotterdam, 3015 CN, Rotterdam, The Netherlands; Department of Neurology, MS Center ErasMS, Erasmus MC, University Medical Center Rotterdam, 3015 GD, Rotterdam, The Netherlands; 1 Department of Immunology, MS Center ErasMS, Erasmus MC, University Medical Center Rotterdam, 3015 CN, Rotterdam, The Netherlands; 1 Department of Immunology, MS Center ErasMS, Erasmus MC, University Medical Center Rotterdam, 3015 CN, Rotterdam, The Netherlands; Department of Neurology, MS Center ErasMS, Erasmus MC, University Medical Center Rotterdam, 3015 GD, Rotterdam, The Netherlands; Neuroimmunology Research Group, Netherlands Institute for Neuroscience, 1105 BA, Amsterdam, The Netherlands

**Keywords:** primary progressive multiple sclerosis, ocrelizumab, CD20^dim^ T cell, B cell, CSF

## Abstract

The anti-CD20 monoclonal antibody ocrelizumab reduces disability progression in primary progressive multiple sclerosis. CD20 is a prototypical B-cell marker; however, subpopulations of CD4^+^ and CD8^+^ T cells in peripheral blood and cerebrospinal fluid also express low levels of CD20 (CD20^dim^). Therefore, direct targeting and depletion of these CD20^dim^ T-cell subpopulations may contribute to the therapeutic effect of ocrelizumab. The aim of this observational cohort study was to compare CD20^+^ B-cell and CD20^dim^ T-cell distributions between peripheral blood and cerebrospinal fluid of ocrelizumab-treated or ocrelizumab-untreated people with primary progressive multiple sclerosis. Ocrelizumab treatment was associated with depletion of circulating B cells and CD20^dim^ CD4^+^ and CD20^dim^ CD8^+^ T cells (*P* < 0.0001, *P* = 0.0016 and *P* = 0.0008, respectively) but, in cerebrospinal fluid, only with lower proportions of B cells and CD20^dim^ memory CD4^+^ T cells (*P* < 0.0001 and *P* = 0.0043, respectively). The proportional prevalence of cerebrospinal fluid CD20^dim^ memory CD8^+^ T cells was not significantly reduced (*P* = 0.1333). Only in cerebrospinal fluid, the proportions of CD20^dim^ cells within CD4^+^ and not CD8^+^ T cells positive for CCR5, CCR6 and CXCR3 were reduced in ocrelizumab-treated participants. The proportion of CD20^dim^ CD4^+^ T cells and abundance of CD4^+^ relative to CD8^+^ T cells in cerebrospinal fluid correlated positively with age (*R* = 0.6799, *P* = 0.0150) and Age-Related Multiple Sclerosis Severity score (*R* = 0.8087, *P* = 0.0014), respectively. We conclude that, in contrast to cerebrospinal fluid CD20^dim^ CD8^+^ T cells, B cells and CD20^dim^ CD4^+^ T cells are reduced in cerebrospinal fluid of people with primary progressive multiple sclerosis with an ocrelizumab-associated depletion of circulating B cells and CD20^dim^ T cells. Therefore, these cells are likely to contribute to the therapeutic effects of ocrelizumab in people with primary progressive multiple sclerosis.

## Teaser

van Puijfelik *et al*. report that not only B cells but also CD20^dim^ CD4^+^ and not CD20^dim^ CD8^+^ memory T cells are reduced in the cerebrospinal fluid of people with primary progressive multiple sclerosis after ocrelizumab-associated depletion of circulating B cells and CD20^dim^ T cells. Therefore, both B and CD20^dim^ T memory cells likely contribute to the therapeutic effects of ocrelizumab.

## Graphical Abstract

**Graphical Abstract:**
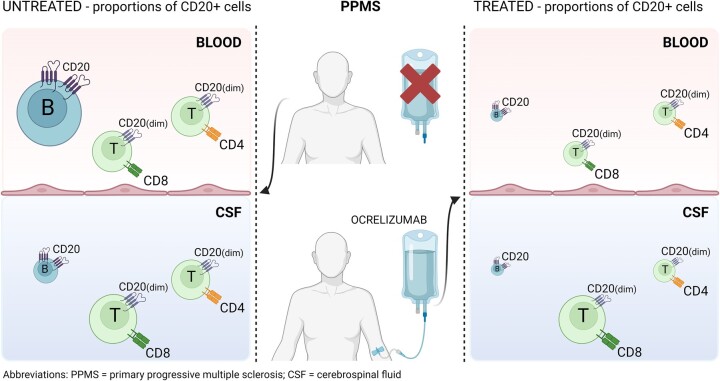

## Introduction

Multiple sclerosis is a chronic inflammatory demyelinating disease of the CNS, in which T cells have a long-established pathogenic role. However, more recent studies on the effectiveness of CD20-targeting therapies in the reduction of disease activity in people with multiple sclerosis highlighted the role of B cells in multiple sclerosis pathogenesis.^1^ Nowadays, the anti-CD20 monoclonal antibody ocrelizumab (OCR) is widely used as a highly effective disease-modifying treatment for relapsing remitting multiple sclerosis and is currently the only disease-modifying treatment that has shown attenuation of disability progression in primary progressive multiple sclerosis.^2-6^

CD20 is a membrane-spanning phosphoprotein strongly expressed on B cells and is widely regarded as a prototypical B-cell–restricted marker.^7^ However, small populations of CD4^+^ and CD8^+^ T cells have been shown to express CD20 at approximately 15-fold lower levels as well.^8^ These CD20^dim^ T cells represent a highly activated population with an increased capacity to produce pro-inflammatory cytokines.^8-11^ Circulating CD20^dim^ T cells are found to be expanded in several chronic inflammatory diseases.^12^ In addition to this, CD20 is enriched on T cells isolated from non-diseased post-mortem human brain tissue, serving as a marker for a subset of CNS-homing T cells.^13,14^ Accordingly, the relative numbers of CD20^dim^ T cells are increased in the circulation and even more so in the CSF and white matter lesions of people with multiple sclerosis.^11,13-17^ There is also evidence that CD20^dim^ T cells are pathogenic in experimental autoimmune encephalomyelitis mice and associated with disease activity in people with relapsing remitting multiple sclerosis and disease severity in people with primary progressive multiple sclerosis.^15,18,19^ Moreover, the depletion of circulating CD20^dim^ T cells has been argued to contribute to OCR treatment efficacy.^19-24^

Compared with rituximab,^25-27^ the effects of OCR on lymphocytes in CSF of people with multiple sclerosis are less known. Interestingly, a recent study showed that the frequencies of circulating or CSF CD20^dim^ T cells were not affected in people with primary progressive multiple sclerosis who received dimethyl fumarate, a disease-modifying treatment commonly used for people with relapsing remitting multiple sclerosis.^15^ Since OCR did and dimethyl fumarate did not show significant effects in clinical trials for people with primary progressive multiple sclerosis, divergent effects on the intrathecal lymphocyte composition could reveal important mechanisms in the modulation of primary progressive multiple sclerosis.^5,28^ Studying lymphocyte fractions in the CSF is especially relevant, since the pathology of primary progressive multiple sclerosis has been argued to be more dependent on compartmentalized inflammatory and degenerative processes and not on CNS recruitment of circulating lymphocytes.^29^ Additionally, phenotypically distinct populations have been reported to patrol the intrathecal compartment in people with advanced progressive multiple sclerosis, and monoclonal antibodies as OCR do not cross the blood–brain barrier.^30^ The aim of our current study was to investigate the association of OCR therapy with both B cells and T cells and particularly CD20^dim^ subsets in the blood and CSF of people with primary progressive multiple sclerosis.

## Methods

### Study design

We performed this research as a part of our longitudinal, prospective and observational cohort study ‘Study to Predict Inflammation and Neurodegeneration in PPMS’ (SPIN-P). In this study, we included adults fulfilling the 2017 McDonald criteria for primary progressive multiple sclerosis.^31^ The only exclusion criterion was a life expectancy of 6 months or less. No other inclusion or exclusion criteria such as age, treatment, Expanded Disability Status Scale score or disease duration were used. people with primary progressive multiple sclerosis were asked to voluntarily participate in this study by donating blood and CSF sample at inclusion and at 1-year follow-up.^32^ The study was approved by the medical ethics committee of the Erasmus Medical Center. All participants provided informed consent. The first participant was included on 12 June 2020. For this research, people with primary progressive multiple sclerosis who underwent a lumbar puncture when treated with OCR were matched with people with primary progressive multiple sclerosis who did not receive OCR, based on age and sex. All included people with primary progressive multiple sclerosis did not receive any other immune-modifying therapy (IMT) in at least 3 months prior to the lumbar puncture.

### Cell isolation and flow cytometry

Peripheral blood mononuclear cells (PBMCs) were isolated from whole blood according to the manufacturer’s instructions with the use of vacutainer CPT® tubes containing sodium heparin (BD Biosciences, Erembodegem, Belgium). CSF of people with primary progressive multiple sclerosis was obtained through lumbar puncture. Cells from CSF were isolated by spinning down at 500 *g* for 10 min. PBMC and CSF samples were taken on the same day and immediately used for phenotyping using conventional or spectral flow cytometry. Due to advancement in laboratory procedures during the course of our study, phenotyping data were acquired using both a BD LSRFortessa™ flow cytometer and a Cytek® Aurora™ spectral analyser (5-laser; 355, 405, 488, 561 and 640 nm). Repeated samples were analysed using the same machine, and the comparability of both approaches was validated. We used a 13 and 37 colour-based panel, respectively, with fluorochrome-labelled monoclonal anti-human antibodies as described in Supplementary Tables 1 and 2. The 13 colour-based flow cytometry was performed using a variety of fluorochrome-labelled monoclonal anti-human antibodies (Supplementary Table 1) as described previously.^33^ All cells were incubated with Human TruStain FcX Fc Receptor Blocking Solution (BioLegend) for 10 min at room temperature in the dark. All pre-titrated antibodies targeting chemokine receptors were added sequentially to the cells re-suspended in 1/5 BSB plus staining buffer (BD Biosciences) with a total volume of 50 µl. These were incubated ranging from 5 to 15 min at room temperature in the dark. After washing, all antibodies with fluorochromes peaking in the UV or violet channel were added sequentially to the cells, which were incubated for 15 min at room temperature in the dark after the last addition. Lastly, after two washing cycles, a master mix of all remaining antibodies was added and incubated for 20 min at room temperature in the dark. Cells were then washed and re-suspended in 150-µl PBS + 0.2% BSA for measurement with the Cytek® Aurora™ using SpectroFlo Software (V3.1.0). The cells were unmixed using single stained PBMCs from a healthy donor as reference controls and a fraction of unstained cells per tissue compartment of each multiple sclerosis donor. Analysis was performed using OMIQ software version 9.5.1 (from Dotmatics; https://www.omiq.ai/). Due to the switch in machines and fluorescent-labelled antibodies, we only compared percentages of positive cells and were not able to use mean fluorescence intensities. To ensure high quality of data, samples with <50 cell events within the analysed gate were censored. Representative plots and the used gating strategy can be found in Supplementary Fig. 1. Cells measured with spectral flow cytometry are represented by open dots in the graphs.

### Clinical outcomes

The percentages of B cells and accompanying T-cell fractions in blood and CSF were correlated to age and Age-Related Multiple Sclerosis Severity (ARMSS) score as parameters relevant for disease progression.^34^

### Statistical analysis

Comparative analyses of differences in baseline characteristics between both groups were performed using the appropriate relevant statistical methods: Fisher’s exact test or Mann–Whiney *U* test. Statistical analyses were performed using GraphPad Prism (version 9.0.0, San Diego, CA, USA) or SPSS (version 28.0.1.0, IBM SPSS Statistics); specific details are given in each figure legend. *P*-values of <0.05 were considered significant.

### Ethical statement

The studies involving human participants were reviewed and approved by the Medical Ethics Committee Erasmus MC (MEC-2014-033). The participants provided their written informed consent to participate in this study.

## Results

### Participants and CSF sampled

A total of 13 people with primary progressive multiple sclerosis treated with OCR were matched to 13 untreated people with primary progressive multiple sclerosis. One person in the OCR-treated group was excluded, since the lumbar puncture was done 454 days after the last dose of OCR. Therefore, we were not able to consider this sample as an OCR-treated or OCR-untreated sample. Relevant clinical characteristics were comparable between the OCR-treated and OCR-untreated group. Particularly, these groups were similar regarding the number of males, age at the time of sampling, age at diagnosis, disease duration and Expanded Disability Status Scale at the time of sampling (Table 1). Within 1 year prior to sampling, none of the OCR-treated people with primary progressive multiple sclerosis and two OCR-untreated people with primary progressive multiple sclerosis displayed inflammatory disease activity, defined as the presence of relapses, gadolinium-enhancing lesions or the presence of a new lesion on follow-up MRI (0% versus 15.4%, respectively; *P* = 0.260). Of the OCR-treated group, six people with primary progressive multiple sclerosis (50%) had disease activity identifiable on MRI in the year before the start of OCR, with one of these people also experiencing clinical relapses. People in the OCR-treated group received a median of 4.5 doses of OCR at the time of sampling (range 2–5). The median number of days since the last dose of OCR at time sampling was 117 (range 14–189). Lastly, besides OCR, there were no substantial differences regarding IMT use prior to the sample collection. In the OCR-treated group, four people with primary progressive multiple sclerosis (33.3%) had previously had pulse corticosteroids, with a median amount of 35.3 months since the stop of this treatment before the sample collection for the current study (range 5.3–99.3 months), and one person (8.3%) had previously had teriflunomide, until 13.5 months before sample collection. In the untreated group, only one person (7.7%) had had prior IMT, namely pulse corticosteroids, until 2.9 months before the current sample collection. The clinical characteristics of included people with primary progressive multiple sclerosis are summarized in Table 1.

**Table 1:** Baseline characteristics

	Untreated (*n* = 13)	OCR-treated (*n* = 12)	*P*
**Male; *n* (%)**	4 (30.8)	5 (41.7)	*P* = 0.440^a^
**Age at sample (years); median (range)**	50.0 (43.2-61.4)	57.6 (37.2-62.0)	*P* = 0.320^b^
**Age at diagnosis (years); median (range)**	47.6 (39.2–56.2)	50.0 (36.1–59.5)	*P* = 0.852^b^
**Disease duration (years, since symptom onset); median (range)**	7.1 (2.0–13.2)	8.1 (2.7–14.1)	*P* = 0.295^b^
**EDSS at sample; median (range)**	4.0 (2.0–6.5)	4.25 (3.5–7.5)	*P* = 0.406^b^
**Prior IMT; *n* (%)**			
**None**	12 (92.3%)	7 (57.8%)	*P* = 0.073^a^
**Pulse corticosteroids**	1 (7.7%)	4 (33.3%)	
**Months since stop of prior IMT; median (range)**	2.9	35.3 (5.3–99.3)	
**Teriflunomide**	0	1 (8.3%)	
**Months since stop of prior IMT; median (range)**	N.A.	13.5	
**Disease activity in year before sample** ^c^ **; *n* (%)**	2 (15.4%)	0 (0%)	*P* = 0.260^a^
**MRI; *n* (%)**	2 (15.4%)	0 (0%)	
**Relapses; *n* (%)**	0 (0%)	0 (0%)	
Disease activity in year before start of OCR*; *n* (%)	N.A.	6 (50%)	
**MRI; *n* (%)**		6 (50%)	
**Relapses; *n* (%)**		1 (8.3%)	
**Doses of OCR at sample; median (range)**	N.A.	4.5 (2–5)	
**Days since last dose of OCR at sample; median (range)**	N.A.	117 (14–189)	

^a^Fisher’s exact.

^b^Mann–Whitney *U*.

^c^Disease activity defined as the presence of relapses, gadolinium-enhancing lesions or the presence of a new lesion on follow-up MRI within 1 year. In cases where no MRI within the year before sample or before start of OCR was performed, we scored the disease activity to be absent.

EDSS, Expanded Disability Status Scale; IMT, immunomodulating treatment; N.A., not applicable; OCR, ocrelizumab.

### CD4^+^ and CD8^+^ memory T cells expressing CD20, CCR5 and CXCR3 are enriched in the CSF of untreated people with primary progressive multiple sclerosis

**Figure 1:**
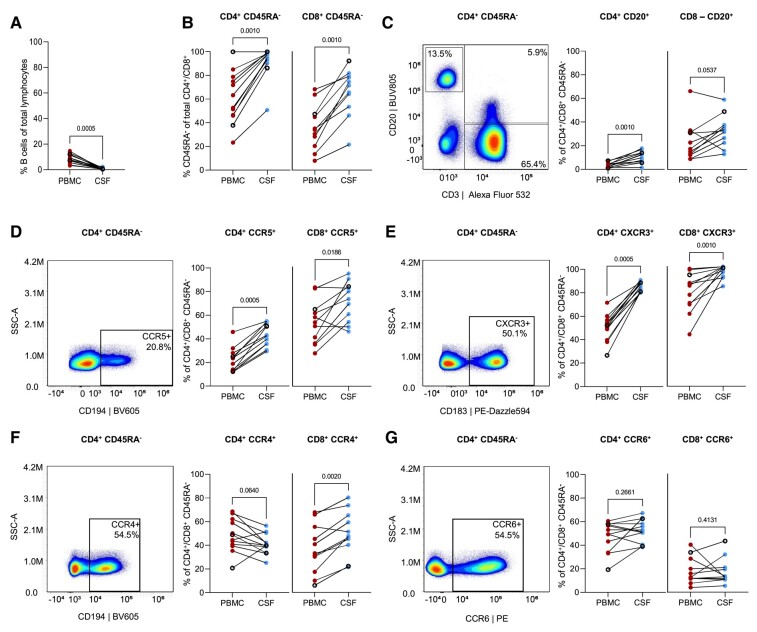
**Relative frequencies and brain-homing phenotypes of CD4^+^ and CD8^+^ T cells in paired PBMC and CSF of untreated people with primary progressive multiple sclerosis.** This figure shows untreated people with primary progressive multiple sclerosis (*n* = 13). (**A**) Percentages of B cells within total lymphocyte population in PBMC and CSF. (**B**) Percentages of memory (CD45RA^−^) cells within the total CD4^+^ (left) or CD8^+^ (right) T-cell pool in PBMC and CSF. (**C–G**) Representative dot plots and percentages of CD20, CCR5, CXCR3, CCR6 and CCR4 within total CD4^+^/CD8^+^ memory T cells of a people with primary progressive multiple sclerosis. Statistical significance was tested using Wilcoxon tests. *P*-values of <0.05 were considered significant. Data acquired through traditional flow cytometry are denoted by solid dots (UNTX: *n* = 10; TX: *n* = 8), while data obtained via spectral flow cytometry are indicated by open dots (UNTX: *n* = 3; TX: *n* = 4). Due to changes in measured markers overtime, the amount of dots may differ per graph. SSC-A, sideward scatter area.

First, we analysed the relative numbers of B cells and both CD4^+^ and CD8^+^ memory (CD45RA^−^) T cells in paired PBMC and CSF samples from people with primary progressive multiple sclerosis without OCR treatment. In particular, we zoomed in on CD45RA^−^ memory T cells for the expression of CSF- and/or brain residency-associated T-cell markers CD20,^13^ CCR5,^35^ CXCR3 and CCR6,^35-38^ as well as CCR4, a more skin-homing and T helper cell–defining marker.^39,40^ We showed low prevalence of B cells and an enrichment of CD4^+^ and CD8^+^ CD45RA^−^ memory T cells in primary progressive multiple sclerosis CSF versus PBMCs (Fig. 1A and B). Phenotypically, compared with PBMC fractions, these CSF memory T cells were characterized by a higher proportion of CD20^+^ (Fig. 1C), CCR5^+^ (Fig. 1D) and CXCR3^+^ T cells (Fig. 1E), yet with a similar abundance of CCR6^+^ T cells (Fig. 1F). CCR4 expression was more abundant on CSF CD8^+^ CD45RA^−^ memory T cells, yet lower on the CD4^+^ fraction, compared with PBMCs (Fig. 1G).

### OCR treatment is associated with reduced frequencies of B cells and CD20^dim^ T cells in the PBMC fraction of people with primary progressive multiple sclerosis

**Figure 2:**
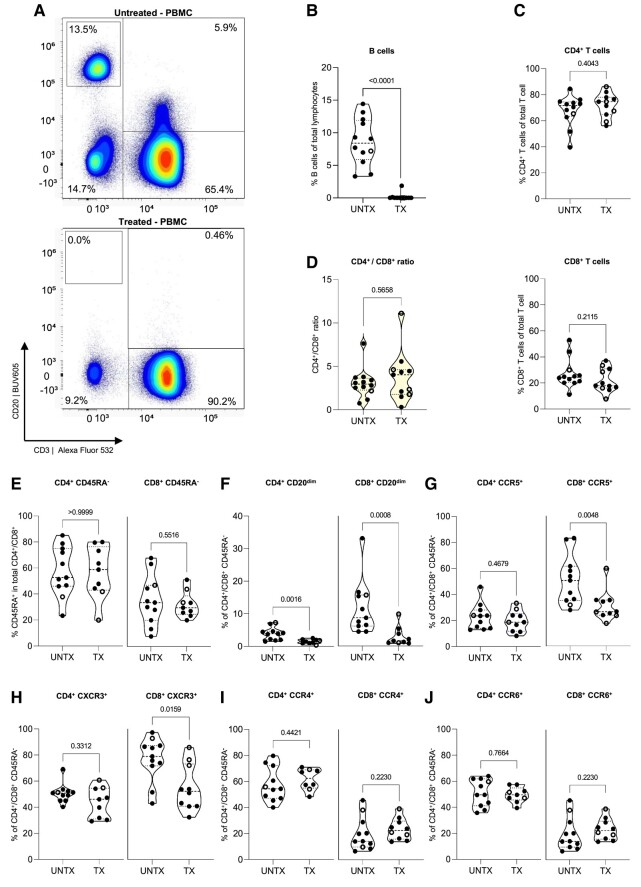
**Frequencies of B-cell and T-cell subsets within the PBMC fraction of untreated and OCR-treated people with primary progressive multiple sclerosis**. (**A**) Representative dot plots showing CD20 versus CD3 in PBMCs of an untreated (top) and OCR-treated (bottom) people with primary progressive multiple sclerosis. (**B**) Violin plot showing the percentage of B cells of total lymphocytes in age- and gender-matched untreated (UNTX: *n* = 13) and OCR treated (TX: *n* = 12) people with primary progressive multiple sclerosis. (**C**) Violin plot showing the percentage of CD4^+^ (top) and CD8^+^ (bottom) of total T cells (**D**) and showing the CD4^+^/CD8^+^ ratio. (**E**) Violin plot showing the percentage of CD4^+^ and CD8^+^ memory (CD45RA^−^) T cells within total lymphocytes. (**F–J**) Violin plot showing the percentage of CD20^dim^, CCR5^+^, CXCR3^+^, CCR4^+^ and CCR6^+^ subsets within CD4^+^ (left) and CD8^+^ (right) memory T cells. Statistical significance was tested using Mann–Whitney *U* tests. *P*-values of <0.05 were considered significant. Each violin plot shows median and quartiles through dotted lines. Data acquired through traditional flow cytometry are denoted by solid dots (UNTX: *n* = 10; TX: *n* = 8), while data obtained via spectral flow cytometry are indicated by open dots (UNTX: *n* = 3; TX: *n* = 4). Due to changes in measured markers overtime, the amount of dots may differ per graph. TX, treated; UNTX, untreated.

**Figure 3:**
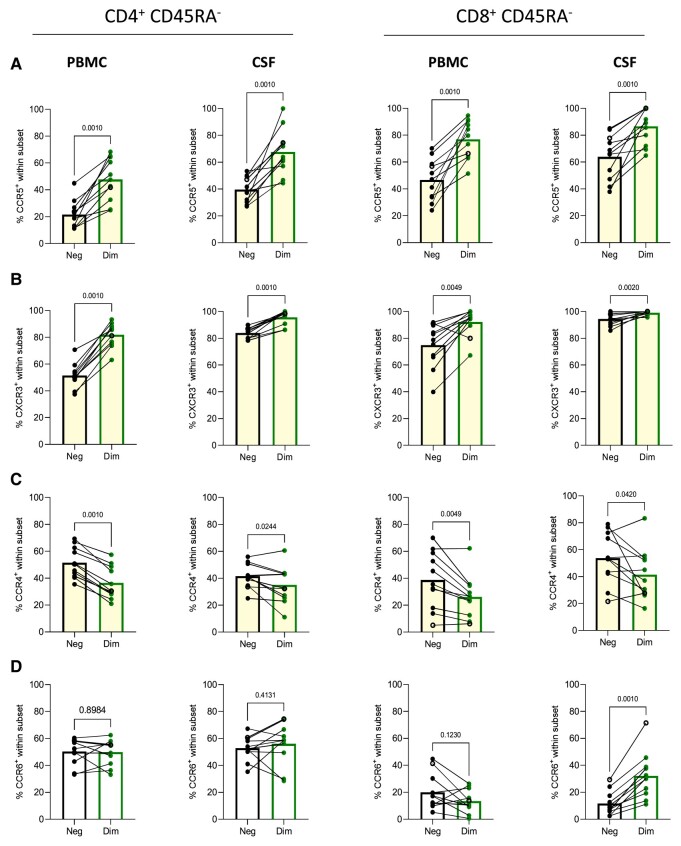
**Expression of brain-homing markers on paired CD20^dim^ versus CD20^neg^ subsets within memory T cells of untreated people with primary progressive multiple sclerosis**. Samples with frequencies of CD20^neg^ and CD20^dim^ CD4^+^ (left panel) or CD8^+^ (right panel) memory (CD45RA^−^) T cells expressing CCR5 (**A**), CXCR3 (**B**), CCR4 (**C**) and CCR6 (**D**) in PBMC and CSF from untreated people with primary progressive multiple sclerosis (*n* = 13). Statistical significance was tested using Wilcoxon tests. *P*-values of <0.05 were considered significant. Data acquired through traditional flow cytometry are denoted by solid dots (UNTX: *n* = 10), while data obtained via spectral flow cytometry are indicated by open dots (UNTX: *n* = 3). Due to changes in measured markers overtime, the amount of dots may differ per graph. dim, CD20^dim^; neg, CD20^neg^; TX, treated; UNTX, untreated.

Next, we compared the distribution of B cells and CD4^+^ and CD8^+^ CD45RA^−^ memory T cells in PBMCs of people with primary progressive multiple sclerosis with versus without OCR treatment. As expected, PBMCs of OCR-treated individuals hardly contained B cells compared with OCR-untreated participants (Fig. 2A and B). The frequencies of both total and CD45RA^−^ memory CD4^+^ and CD8^+^ T cells and their ratios were not different between the treated and untreated group (Fig. 2C–E). OCR-treated individuals did show lower proportions of CD20^dim^ cells within both the CD4^+^ and CD8^+^ CD45RA^−^ memory T cell fractions (Fig. 2F). No decrease was seen in CD20-negative T cells within the CD4^+^ and CD8^+^ CD45RA^−^ memory PBMC compartments (Supplementary Fig. 2), nor a significant correlation of CD20^dim^ T cell proportions with time since infusion (Supplementary Fig. 3). This lower CD20^dim^ proportion coincided with lower proportions of CD8^+^ and not so much CD4^+^ CD45RA^−^ memory T cells being CCR5^+^ or CXCR3^+^ (Fig. 2G and H). The distribution of CCR6^+^ and CCR4^+^ fractions within CD4^+^ and CD8^+^ CD45RA^−^ memory T cells was similar between both treatment groups (Fig. 2I and J). In contrast to CCR6 and CCR4, CD20^dim^ T cells showed higher levels of CCR5 and CXCR3 (Fig. 3A and B) compared with CD20-negative counterparts, which was in line with previous work.^15^ Accordingly, we found a prominent loss of CD20^dim^ T cells within specifically the CXCR3^+^ and CCR5^+^ T-cell fractions in the OCR-treated group (Supplementary Fig. 4). This shows that indeed the lowering of CXCR3^+^ and CCR5^+^ subsets within CD45RA^−^ memory CD8^+^ T cells correlates with a loss of CD20^dim^ cells in the treated group. These data indicate that in PBMCs of people with primary progressive multiple sclerosis, OCR treatment profoundly depletes B cells as well as CD20^dim^ CD4^+^ and CD8^+^ T cells and that this depletion contributes to an overall loss of CXCR3^+^ and CCR5^+^ T cells especially in the CD8^+^ memory pool.

### B-cell and CD20^dim^ CD4^+^ memory T-cell fractions are significantly reduced in the CSF from OCR-treated people with primary progressive multiple sclerosis

**Figure 4:**
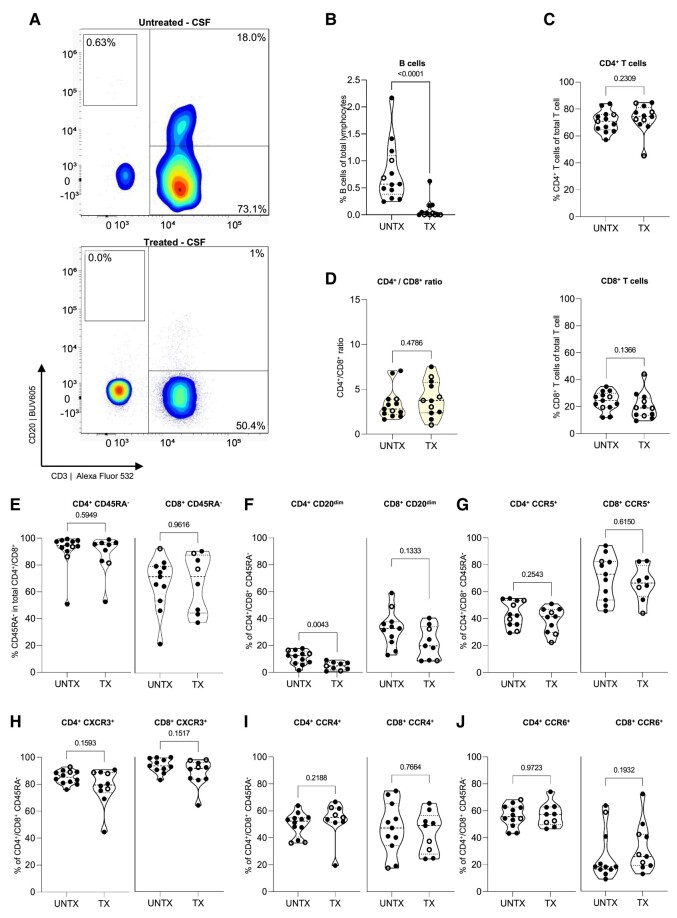
**Frequencies of B-cell and T-cell subsets with a brain-homing phenotype in the CSF of people with primary progressive multiple sclerosis with and without OCR treatment**. (**A**) Representative dot plots showing CD20 versus CD3 in CSF of an untreated (top) and OCR-treated (bottom) people with primary progressive multiple sclerosis. (**B**) Violin plot showing the percentage of B cells of total lymphocytes in age- and gender-matched untreated (UNTX: *n* = 13) and OCR-treated (TX: *n* = 12) people with primary progressive multiple sclerosis. (**C**) Violin plot showing the percentage of CD4^+^ (top) and CD8^+^ (bottom) of total T cells (**D**) and showing the CD4^+^/CD8^+^ ratio. (**E**) Violin plot showing the percentage of CD4^+^ and CD8^+^ memory (CD45RA^−^) T cells within total lymphocytes. (**F–J**) Violin plot showing the percentage of CD20^dim^, CCR5^+^, CXCR3^+^, CCR4^+^ and CCR6^+^ subsets within CD4^+^ (left) and CD8^+^ (right) memory T cells. Statistical significance was tested using Mann–Whitney *U* tests. *P*-values of <0.05 were considered significant. Each violin plot shows median and quartiles through dotted lines. Data acquired through traditional flow cytometry are denoted by solid dots (UNTX: *n* = 10; TX: *n* = 8), while data obtained via spectral flow cytometry are indicated by open dots (UNTX: *n* = 3; TX: *n* = 4). Due to changes in measured markers overtime, the amount of dots may differ per graph. TX, treated; UNTX, untreated.

In the CSF of people with primary progressive multiple sclerosis, OCR treatment was associated with a lower proportion of B cells (Fig. 4A and B) but a similar distribution of total and memory T cells for both the CD4^+^ and CD8^+^ population (Fig. 4C–E). OCR-treated people with primary progressive multiple sclerosis had a lower proportion of CD20^dim^ cells within the CSF CD45RA^−^ memory CD4^+^ T-cell pool, which was not significantly lower within the CSF CD8^+^ CD45RA^−^ memory T-cell pool (Fig. 4F). In contrast to PBMC (Fig. 3), there was no association of OCR treatment with both total CD4^+^ and CD8^+^ memory T-cell proportions positive for CCR5 (Fig. 4G) or CXCR3 (Fig. 4H) in CSF. Also, the presence of CCR6^+^ and CCR4^+^ CD45RA^−^ memory T cells in the CSF did not differ in the OCR treatment group (Fig. 4I and J). However, CCR5^+^, CXCR3^+^, CCR6^+^ and CCR4^+^ cells within the CD4^+^ CD45RA^−^ memory T-cell pool of the CSF of OCR-treated people with primary progressive multiple sclerosis were all depleted for CD20^dim^ T cells, which was not the case for the CD8^+^ memory T-cell pool (Supplementary Fig. 4). These results suggest that for CD4^+^ CD45RA^−^ memory T cells, the presence of CD20^dim^ fractions in the CSF of people with primary progressive multiple sclerosis (Fig. 1) is more closely associated with the proportions of its PBMC counterparts and the depletion, thereof, than for CD8^+^ CD45RA^−^ memory T cells.

### The presence of CD20^dim^ CD4^+^ memory T cells in the CSF is associated with higher age and ARMSS score in treatment-naive people with primary progressive multiple sclerosis

**Figure 5:**
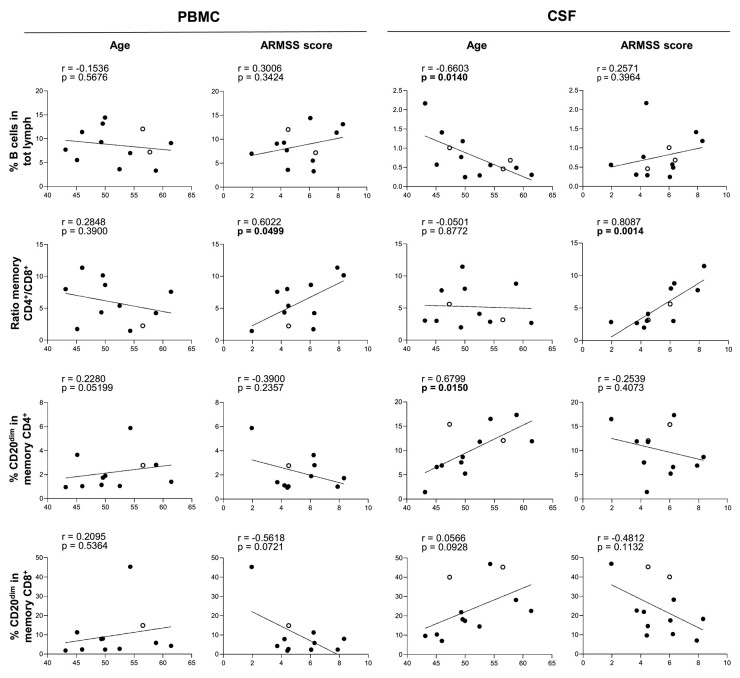
**Correlations between the presence of B-cell and CD20^dim^ T-cell subsets with age and ARMSS score in untreated people with primary progressive multiple sclerosis**. The percentages of B cells within total lymphocytes, CD4^+^/CD8^+^ memory (CD45RA^−^) T-cell ratios as well as CD20^dim^ cells within the CD4^+^ and CD8^+^ memory (CD45RA^−^) T-cell pool in both PBMC (left) and CSF (right) were associated with age (years) and ARMSS scores of untreated people with primary progressive multiple sclerosis (*n* = 13). Statistical significance was tested using Pearson *r* tests. *P*-values of <0.05 were considered significant and are indicated using a bold font. Data acquired through traditional flow cytometry are denoted by solid dots (UNTX: *n* = 10), while data obtained via spectral flow cytometry are indicated by open dots (UNTX: *n* = 3). Due to changes in measured markers overtime, the amount of dots may differ per graph.

To explore the clinical relevance of our findings, we correlated the proportions of PBMC and CSF B cells as well as CD20^dim^ CD4^+^ and CD20^dim^ CD8^+^ CD45RA^−^ memory T cells to age and ARMSS score in untreated people with primary progressive multiple sclerosis. Both a high age and disability score are important predictors of the development of progressive multiple sclerosis.^29^ In accordance with earlier findings in healthy donors,^41^ the percentage of CSF B cells negatively correlated with age, while the frequency of CSF CD20^dim^ CD4^+^ but not CD8^+^ memory T cells correlated positively with age (Fig. 5). In addition, in both PBMCs and CSF of untreated people with primary progressive multiple sclerosis, a relative over-abundance of CD4^+^ compared with CD8^+^ CD45RA^−^ memory T cells associated with a higher ARMSS score. No significant correlations were found in OCR-treated participants (Supplementary Fig. 5).

## Discussion

Here, we showed that treatment of people with primary progressive multiple sclerosis with OCR not only depletes both B cells and CD20^dim^ T cells in the PBMC fraction but is also associated with a significant reduction of total B cells and especially CD4^+^ CD20^dim^ CD45RA^−^ memory T cells in the CSF. In sharp contrast to PBMC fraction, the proportional prevalence of CD20^dim^ memory CD8^+^ T cells and their brain–residency-associated chemokine receptor profile in the CSF was only marginally lower in OCR-treated people with primary progressive multiple sclerosis. Since specifically the kinetics of CSF recruitment of B cells and CD20^dim^ CD4^+^ T cells are affected by OCR, we conclude that these cell types are more likely to contribute to the therapeutic effects of OCR in people with primary progressive multiple sclerosis compared with CD8^+^ T cells. The positive correlation of CD20^dim^ CD4^+^ and not CD8^+^ memory T-cell presence with both a higher age and ARMSS score, two core hallmarks of progressive multiple sclerosis, supports the latter hypothesis.

The origin and function of CD20^dim^ T cells have been a topic of debate. Although trogocytosis was recently shown as a mechanism for T cells to acquire CD20 in the context of experimental autoimmune encephalomyelitis,^18^ the expression of CD20 mRNA by human brain CD4^+^ and CD8^+^ T cells suggests this molecule to be part of the CNS–residency transcriptional programme.^14^ This programme is also characterized by expression of CCR5 and CXCR3.^35,37,42,43^ CXCR3 and its ligands CXCL9, CXCL10 and CXCL11 have previously been described in the context of multiple sclerosis and even as potential therapeutic targets.^44^ Increased CXCR3 expression on CD4^+^ lymphocytes in peripheral blood has been correlated with multiple sclerosis relapses.^45^ Within multiple sclerosis lesions, lymphocytic cells express CXCR3 in nearly all perivascular inflammatory infiltrates.^35,46,47^ Its ligand, CXCL9, was shown to act as a homing chemokine in micro-vascular endothelial cells and astrocytes from the human brain, while CXCL10 and CXCL11 are induced in response to inflammatory stimuli.^48^ Moreover, CXCL10 has shown a significant correlation with the expression of CXCR3 on CSF CD4^+^ T cells.^37,49^ In people with relapsing remitting multiple sclerosis, a higher concentration of CXCL10 in the CSF has been reported.^50^ Lastly, CD8^+^ CD20^dim^ T cells in the CSF also express higher levels of CCR6. This might suggest that these T cells travel between CSF and brain parenchyma rather than from the peripheral blood compartment to CSF, since CCR6 expression has previously been associated with the transmigration across the choroid plexus.^38^ The precise role of these chemokines and their receptors on the exact kinetics and timing of recruitment of T cells into the CNS of people with primary progressive multiple sclerosis remains to be elucidated.

The disease process of progressive multiple sclerosis is not fully understood but is characterized by compartmentalized inflammation and loss of axons behind a closed blood–brain barrier.^29^ Despite a plethora of therapies for relapsing remitting multiple sclerosis, no efficacious treatment for primary progressive multiple sclerosis was available until the seminal phase 3 trial with OCR.^3^ This trial drew a focus on the B cell as a contributor to progressive multiple sclerosis. Indeed, in advanced multiple sclerosis, we and others showed B cell infiltrates and intrathecal antibody production to remain a hallmark of multiple sclerosis pathobiology.^51^ A reduction of PBMC B cells and CD20^dim^ T cells has been demonstrated in OCR-treated people with relapsing remitting multiple sclerosis and primary progressive multiple sclerosis,^21-23,52^ yet effects of this therapy on intrathecal cell populations are most relevant in the context of compartmentalized progressive multiple sclerosis. Interaction of lymphocyte populations in the perivascular space and meninges has been suggested to rather drive lesion expansion and associated disability progression.^53^ Similar to the CSF T cells in the current study, perivascular CD4^+^ and CD8^+^ T cells express tissue residency-associated programmes and phenotypic markers, including CXCR3 and CCR5.^14,54,55^ Interestingly, for CD4^+^ T cells, these programmes share a substantial overlap with phenotypic markers of peripheral helper T cells prone to interact with B-cell populations locally at sites of inflammation, including expression of CCR2, CCR5 and PD-1.^56^ These specific T-cell populations could be instrumental in the association of local antibody-secreting cell formation with a higher local CD4^+^/CD8^+^ T-cell ratio in the context of multiple sclerosis white matter lesions.^51^ Although CD8^+^ T cells are most prevalent in multiple sclerosis white matter lesions and even infiltrate the parenchyma, their effector profile in advanced multiple sclerosis remains uncertain.^14,54^ As recently suggested by Ostkamp *et al*.,^57^ trafficking of CD8^+^ T cells with tissue resident memory T-cell characteristics between perivascular space and CSF could be a dynamic process within the borders of the CSF. Alternatively, the swiftness of CSF repopulation after CD20 treatment by peripheral CD20^dim^ cells might differ between CD4^+^ and CD8^+^ T cells. Nevertheless, our findings show that, in contrast to CD4^+^ cells, a profound depletion of circulating CD20^dim^ CD8^+^ T cells does not affect the phenotypic composition of CD8^+^ T cells within the CSF for the markers investigated. This does not exclude a role of CNS resident CD8^+^ T cells in primary progressive multiple sclerosis^58^ yet does not support a clear association of this subset with the therapeutic effects of OCR in primary progressive multiple sclerosis.

A higher age and higher disability score are two extensively consolidated predictors of progressive disease.^29^ Therefore, the effect of age and disability on underlying immunological mechanisms is relevant to understand the immunological nature of progressive disease. The negative correlation between the percentage of B cells in the CSF and age that we found in people with primary progressive multiple sclerosis is likely a consequence of the significant decrease of the number and percentages of B cells with age, which has been extensively described in previous studies.^59-63^ Our study also supports previous observations that the percentage of CD20^dim^ T cells increases with age^64^ and reports an age-associated expansion of specifically the CD20^dim^ CD4^+^ and not CD8^+^ CD20^dim^ memory T cells. In this line, the association of a relative abundance of CSF CD4^+^ compared with CD8^+^ memory T cells with a higher ARMSS score—a powerful method for measuring the relative severity of disability in multiple sclerosis—^34^ provides further support to our hypothesis that this expansion could be a contributor to progressive disease.

In the context of our study, it is important to acknowledge certain limitations. First, we were able to analyse a relatively limited number of participants with primary progressive multiple sclerosis. Second, the presence of OCR in the circulation of treated people with primary progressive multiple sclerosis could possibly mask the CD20 epitope recognized by the 2H7 clone that we used for cytometric detection in this study.^19^ However, Shinoda *et al*.^19^ showed a similar reduction of cells comparing stainings with the anti-CD20 2H7 clone and an intracellular stained CD20 (clone: 1412) after treatment with OCR, indicating that these cells were genuinely depleted rather than merely masked in detection. Additionally, therapeutic antibodies such as OCR induce antibody-dependent cellular and complement-dependent cytotoxicity rapidly and have a terminal half-life of 28 days.^65,66^ Combined with the long time between OCR infusion and blood sampling, a reduction of CD20-positive B and T cells is most likely. Moreover, there is evidence suggesting OCR penetrates the CSF poorly, as has been shown for the anti-CD20 monoclonal antibody rituximab,^67^ which makes covering of the CD20 epitope intrathecally unlikely. Third, an indirect effect of OCR on circulating CD20^dim^ T cells via B-cell depletion cannot be excluded. B-cell depletion was found to change the immune cell profile in multiple sclerosis.^68^ Therefore, the depletion of B cells might have a significant effect on the T-cell compartment. Although we cannot exclude this mechanism, no reductions of CD20-negative T cells were observed.

In conclusion, the use of OCR therapy is associated with the reduction of B cells and also a decreased presence of particularly CD4^+^ CD20^dim^ T cells in the CSF of people with primary progressive multiple sclerosis. Given that CD20^dim^ T cells display characteristics linked to CNS infiltration, their depletion from the PBMC fraction could potentially play a role as mediators of the effectiveness of OCR treatment for people with primary progressive multiple sclerosis. Lastly, a higher CD4^+^/CD8^+^ memory T-cell ratio was associated with a higher ARMSS score in blood and CSF but not in OCR-treated individuals, further supporting the putative benefit of preventing the accumulation of CD20^dim^ CD4^+^ memory T cells into the CSF through life even of people with the progressive form of multiple sclerosis by treatments such as OCR.

## Supplementary Material

fcae021_Supplementary_Data

## Data Availability

The data presented in this study are available upon reasonable request.
