# Supplementary material for: Ocrelizumab associates with reduced cerebrospinal fluid B and CD20dim CD4+ T cells in primary progressive multiple sclerosis
Source: Brain Commun. 2024 Jan 29;6(1):fcae021. doi: 10.1093/braincomms/fcae021 (PMC10881107; doi:10.1093/braincomms/fcae021)
Supplement: fcae021_Supplementary_Data [file fcae021_supplementary_data.pdf]

Supplementary Information File

Ocrelizumab associates with reduced cerebrospinal fluid B and CD20^{dim} CD4^{+} T cells in primary progressive multiple sclerosis

Fabiënne van Puijfelik, Katelijn M. Blok, Romy A.M. Klein Kranenbarg, Jasper Rip, Janet de Beukelaar, Annet F. Wierenga-Wolf, Beatrijs Wokke, Marvin M. van Luijn, Joost Smolders

Marker	Clone	Fluorochrome	Company
CD3	SK7	BV786	BD Biosciences
CD4	OKT4	BV605	Biolegend
CD8	SK1	FITC	BD Biosciences
CD20	2H7	AF700	BD Biosciences
CD45RA	HI100	APC-H7	BD Biosciences
CD183	G025H7	BV421	Biolegend
CD194	L291H4	PE-Dazzle	Biolegend
CD195	2D7	BV711	Biolegend
CD196	G024E3	PE	Biolegend
Fixable viability dye	N.A.	eFluor 506	eBioscience

Supplementary Table 1. Antibodies used for conventional flow cytometry.

Marker	Clone	Fluorochrome	Company
CD3	UCHT1	AF532	Life Technologies
CD4	SK3	YG584 cFluor	Cytek
CD8	SK1	V547 cFluor	Cytek
CD20	2H7	BUV805	BD Biosciences
CD45RA	HI100	BUV395	BD Biosciences
CD183	G025H7	PE-Dazzle 594	Biolegend
CD194	L291H4	BV605	Biolegend
CD195	J418F1	BV711	Biolegend
CD196	G034E3	PE	Biolegend
Zombie Viability	n.a.	Zombie NIR	Biolegend

Supplementary Table 2. Antibodies used for spectral cytometry.

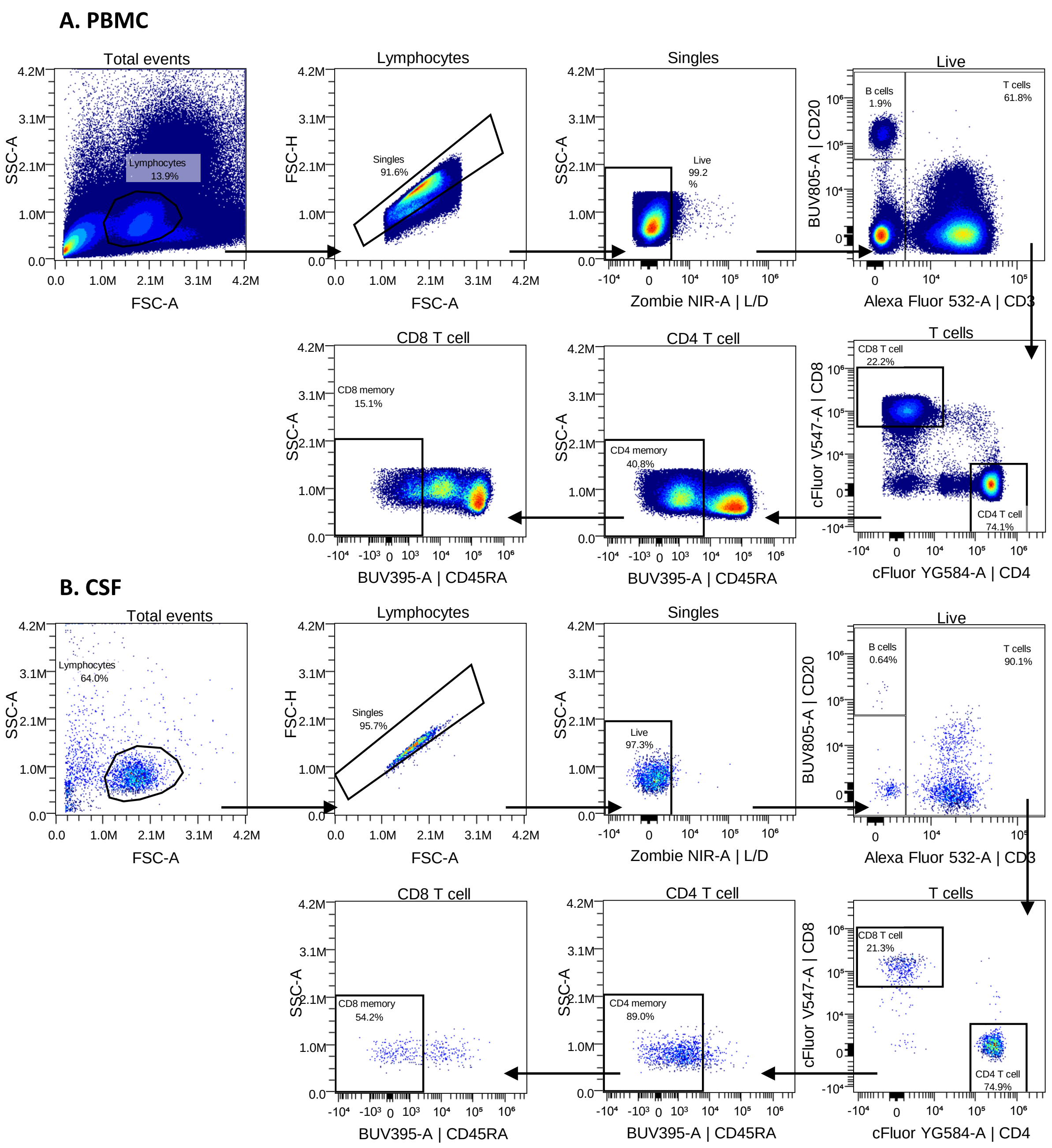

Supplementary Figure 1: Representative dotplots showing gating strategy. Dotplots showing gating strategy up until memory T cell subsets in PBMC (A) and CSF (B) of an untreated person with PPMS.

Abbreviations: PBMC = Peripheral Blood Mononuclear Cell; CSF = cerebrospinal fluid.

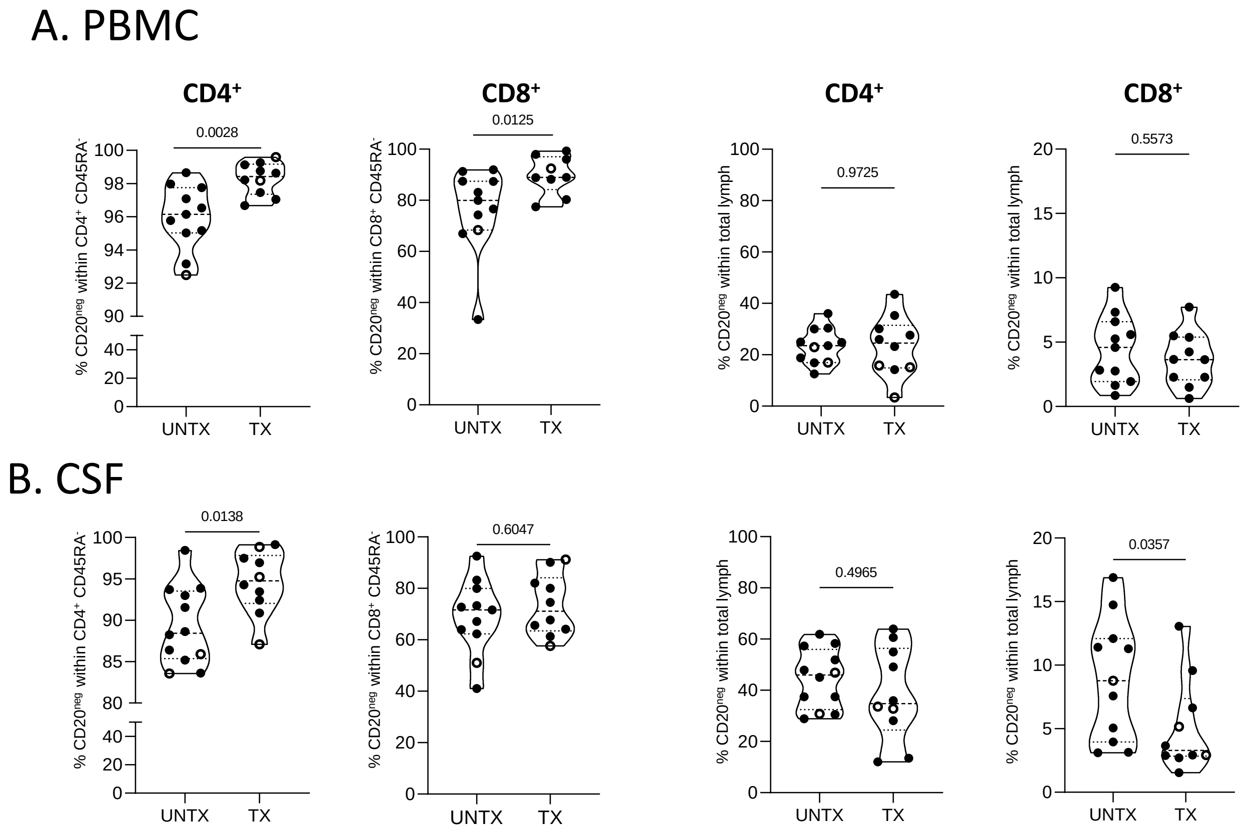

Supplementary Figure 2: CD45RA$^-$ CD20^{neg} T cell ratio within total CD4$^+$/CD8$^+$ CD45RA$^-$ and total T cells in untreated vs treated in PBMCs and CSF. Untreated (UNTX, n=13) and OCR treated (TX, n=12). (A) CD20^{neg} ratio calculated within total CD4$^+$/CD8$^+$ CD45RA$^-$ (left panel) and total T cell (right panel) in PBMCs. (B) CD20^{neg} ratio calculated within total CD4$^+$/CD8$^+$ CD45RA$^-$ (left panel) and total T cell (right panel) in CSF. Statistical significance was tested using Mann-Whitney tests. P-values of < 0.05 were considered significant. Each violin plot shows median and quartiles through dotted lines. Data acquired through traditional flow cytometry is denoted by solid dots (UNTX; n= 10, TX; n=8), while data obtained via spectral flow cytometry is indicated by open dots (UNTX; n= 3, TX; n=4). Due to changes in measured markers overtime the amount of dots may differ per graph.

Abbreviations: PBMC = Peripheral Blood Mononuclear Cell; CSF = cerebrospinal fluid; Lymph = lymphocytes.

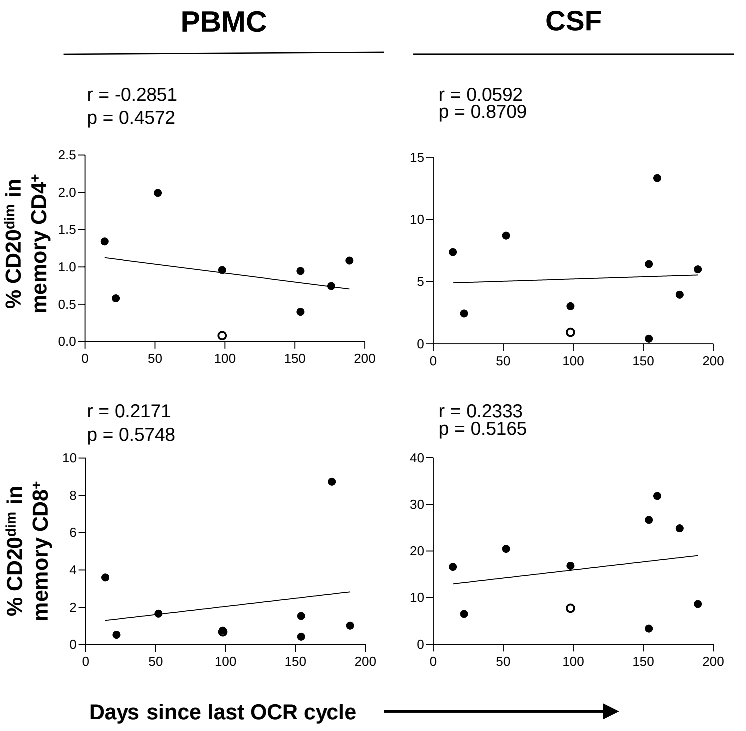

Supplementary Figure 3: The proportions of CD20^{dim} CD4^{+} and CD8^{+} CD45RA^{-} memory T cells in PBMC and CSF of OCR-treated PPMS relative to number of days since ocrelizumab infusion. The percentages of CD20^{dim} within memory (CD45RA^{-}) CD4^{+} and CD8^{+} T cell pool in both PBMC (left) and CSF (right) were associated with days since last OCR cycle of OCR-treated people with primary progressive multiple sclerosis (n=12). Statistical significance was tested using Pearson r tests. P-values of < 0.05 were considered significant. Data acquired through traditional flow cytometry is denoted by solid dots (TX; n=8), while data obtained via spectral flow cytometry is indicated by open dots (TX; n=4). Due to changes in measured markers overtime the amount of dots may differ per graph.
Abbreviations: PBMC = Peripheral Blood Mononuclear Cell; CSF = cerebrospinal fluid; OCR = ocrelizumab.

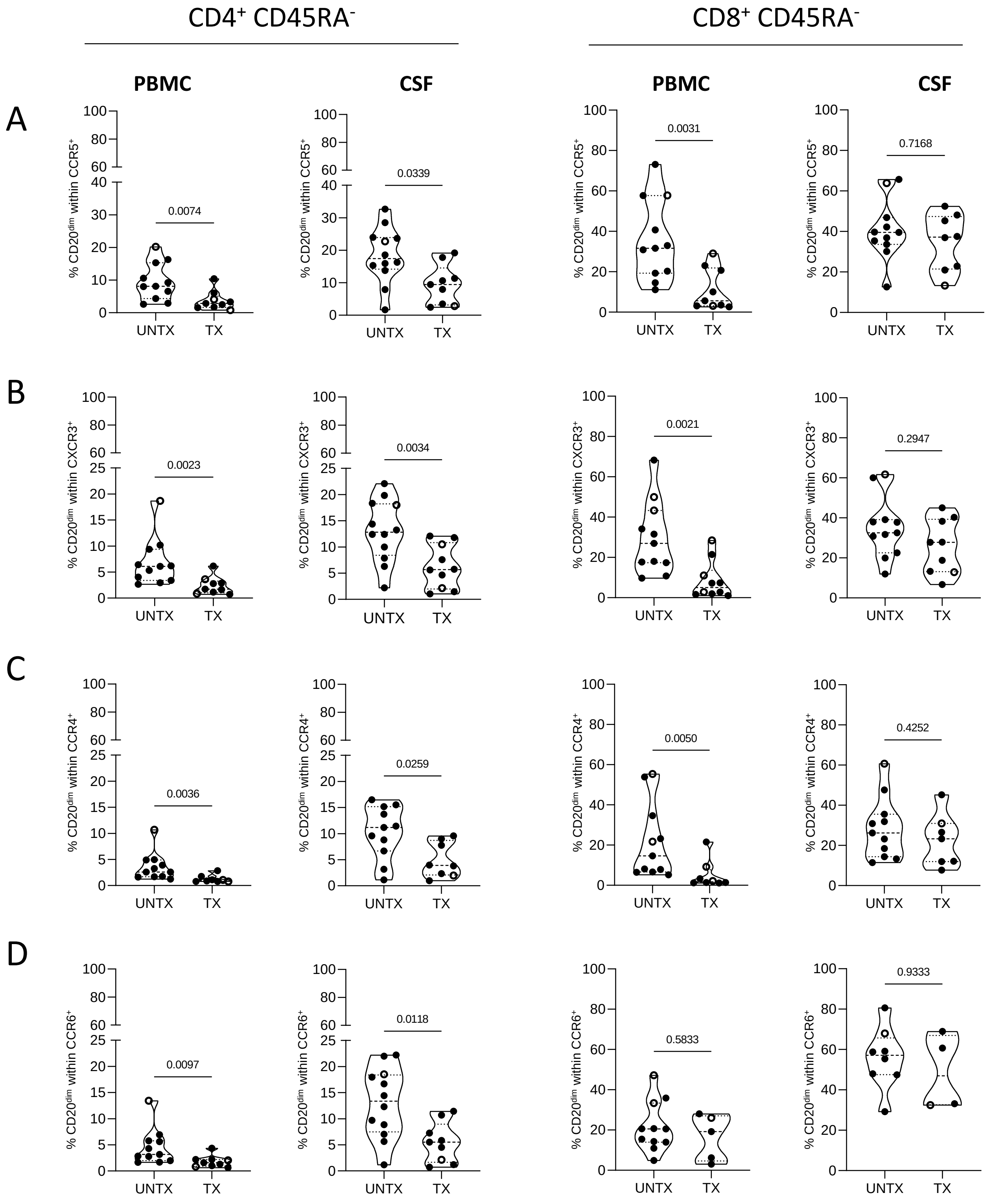

Supplementary Figure 4: The proportion of CD20dim T cells within distinct CD4+ and CD8+ memory subsets in untreated and OCR-treated pwPPMS. Cohort of untreated (UNTX) people with primary progressive multiple sclerosis (pwPPMS) (n=13) and treated (TX) pwPPMS (n=12). Quantitative violin plots comparing UNTX and TX cohorts showing the proportion of $CD20^{dim}$ cells within $CD4^+$ (left) and $CD8^+$ (right) memory ($CD45RA^-$) populations expressing CCR5 (A), CXCR3 (B), CCR4 (C) and CCR6 (D) in both PBMC and CSF. Statistical significance was tested using Mann-Whitney tests. P-values of < 0.05 were considered significant. Each violin plot shows median and quartiles through dotted lines. Data acquired through traditional flow cytometry is denoted by solid dots (UNTX; n= 10, TX; n=8), while data obtained via spectral flow cytometry is indicated by open dots (UNTX; n= 3, TX; n=4). Due to changes in measured markers overtime the amount of dots may differ per graph.

Abbreviations: PBMC = Peripheral Blood Mononuclear Cell; CSF = cerebrospinal fluid.

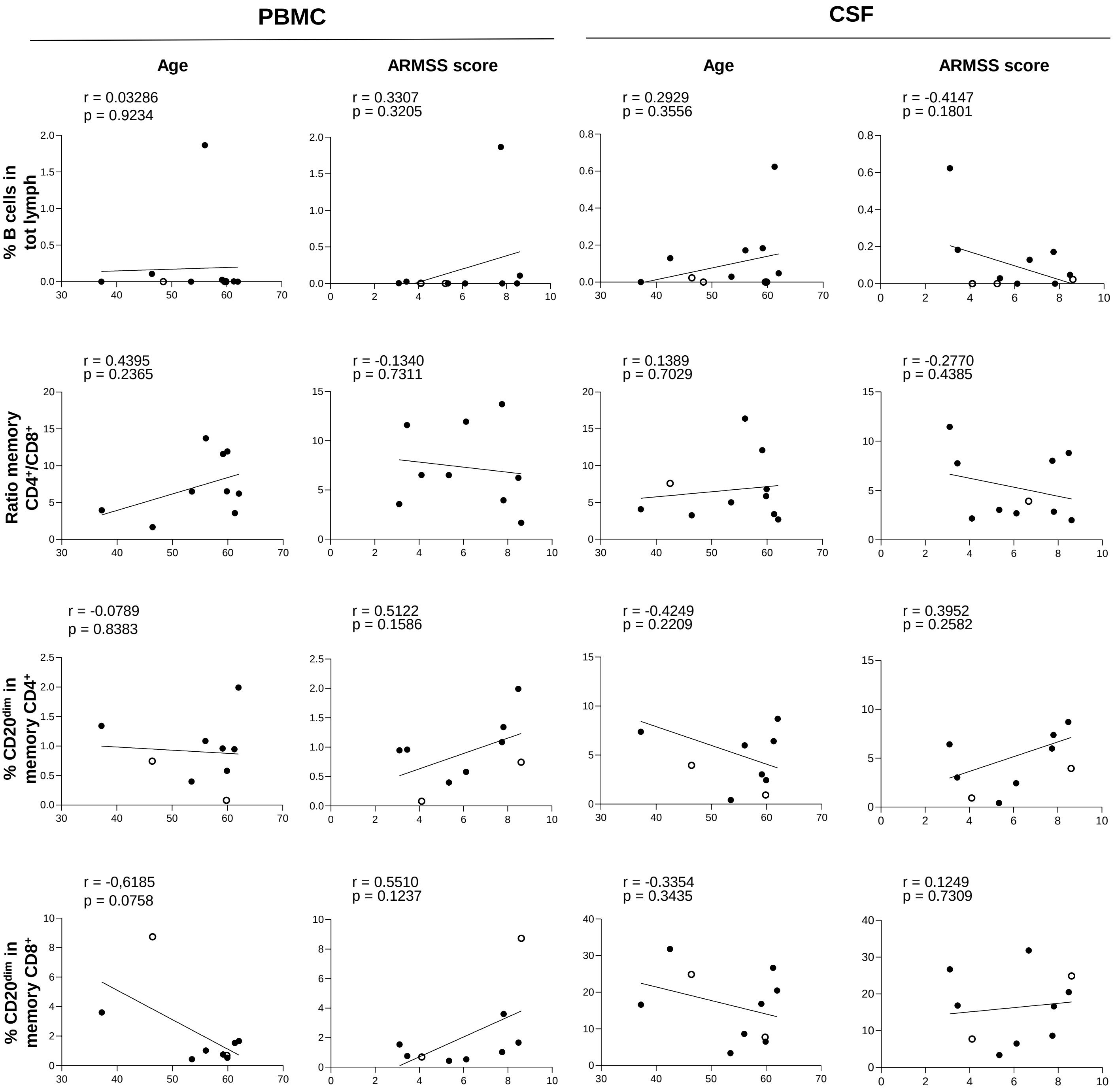

Supplementary Figure 5: Correlations between the presence of B cells and CD20^{dim} T-cell subsets with age and ARMSS score in OCR treated pwPPMS. The percentages of B cells within total lymphocytes, CD4^{+} /CD8^{+} memory (CD45RA^{-}) T-cell ratios as well as CD20^{dim} cells within the CD4^{+} and CD8^{+} memory (CD45RA^{-}) T cell pool in both PBMC (left) and CSF (right) were associated with age (years) and Age Related Multiple Sclerosis Severity (ARMSS) scores of OCR-treated people with primary progressive multiple sclerosis (n=12). Statistical significance was tested using Pearson r tests. P-values of < 0.05 were considered significant and are indicated using a bold font. Data acquired through traditional flow cytometry is denoted by solid dots (TX; n=8), while data obtained via spectral flow cytometry is indicated by open dots (TX; n=4). Due to changes in measured markers overtime the amount of dots may differ per graph.

Abbreviations: PBMC = Peripheral Blood Mononuclear Cell; CSF = cerebrospinal fluid.
